# Complete chloroplast genome sequence of *Scurrula notothixoides* (Loranthaceae): a hemiparasitic shrub in South China

**DOI:** 10.1080/23802359.2018.1471366

**Published:** 2018-05-11

**Authors:** Lang-Xing Yuan, Jian-Hua Wang, Chao-Rui Chen, Kun-Kun Zhao, Zhi-Xin Zhu, Hua-Feng Wang

**Affiliations:** Hainan Key Laboratory for Sustainable Utilization of Tropical Bioresources, Institute of Tropical Agriculture and Forestry Hainan University, Haikou, China

**Keywords:** *Scurrula notothixoides*, Illumina sequencing, plastome, Loranthaceae, phylogenetic analysis, Santalales

## Abstract

*Scurrula notothixoides* (Loranthaceae) is a hemiparasitic shrub distributed in forest margins of Southeast Asian countries. Here, we report and characterize the complete plastid genome sequence of *S. notothixoides* in an effort to provide genomic resources useful for the phylogenetic studies for Santalales. The complete plastome is 123,810 bp in length and contains the typical structure and gene content of angiosperm plastomes, including two inverted repeat (IR) regions of 23,101 bp, a large single copy (LSC) region of 71,448 bp and a small single copy (SSC) region of 6160 bp. The plastome contains 88 genes, consisting of 61 unique protein-coding genes, 23 unique tRNA genes and four unique rRNA genes. The overall A/T content in the plastome of *S. notothixoides* is 62.7%. Phylogenetic analyses were performed using the entire plastome, including spacers, introns, etc. and we recovered that *S. notothixoides* and *Taxillus sutchuenensis* was closely related. The complete plastome sequence of *S. notothixoides* will provide a useful resource for the phylogenetic studies for Santalales.

*Scurrula notothixoides* (Hance) Danser (Loranthaceae) is a hemiparasitic shrub distributed in forest margins of South Guangdong, Hainan of China and Vietnam (Qiu and Gilbert 2003). Recorded hosts for this species include *Citrus aurantium, Cordia dichotoma*, *Euodia lepta*, *Hibiscus tiliaceus*, *Melastoma sp.* Here, we report and characterize the complete plastome of *S. notothixoides* (GenBank accession number: MH220878, this study) based on Illumina paired-end sequencing data. This is the first report of a complete plastome for the genus *Scurrula* and Loranthaceae. Furthermore, we analysed the phylogenetic relationships of *S. notothixoides* within Santalales based on the complete chloroplast genomes to provide baseline data for the phylogenetic studies for Santalales.

In this study, *S. notothixoides* was sampled from Baoting in Hainan province of China. A voucher specimen (H.-F. Wang et al. B126) was deposited in the herbarium of the Institute of Tropical Agriculture and Forestry, Hainan University, Haikou, China. The modified CTAB method of Doyle and Doyle (1987) was used to extract total genomic DNA from leaves quickly frozen with dry ice. An Illumina library kit was applied to an Illumina flow cell for cBOT cluster generation. Sequencing was performed on an Illumina HiSeq instrument. Paired-end, 150 bp reads were sequenced using an Illumina HiSeq 2500 platform at the Guangzhou Novel-seq Biotechnology Co., Ltd. (Guangzhou, China). Reads were trimmed and those with >10% Ns or with >10% low quality (*Q* ≤ 5) bases were filtered using NGSQC-Toolkit v2.3.3 (Patel and Jain 2012). Cleaned reads were assembled against the plastome of *Taxillus sutchuenensis* (GenBank Accession number: NC_036307.1) (Li et al. 2017) using MITO bim v1.8 (Hahn et al. 2013). Each sample’s depth is 40× in this study.

The plastome was annotated using Geneious R8.0.2 (Biomatters Ltd., Auckland, New Zealand) against the plastome of *Taxillus sutchuenensis* (GenBank accession number: NC_036307.1). The annotation was corrected with DOGMA (Wyman et al. 2004). A circular plastome map was generated using OGDRAW (http://ogdraw.mpimp-golm.mpg.de/) (Lohse et al. 2013).

The plastome of *S. notothixoides* was found to possess a total length 123,810 bp with the typical quadripartite structure of angiosperms, containing two inverted repeats (IRs) of 23,101 bp separated by a large single copy (LSC) region and a small single copy (SSC) region of 71,448 and 6160 bp, respectively. The plastome was found to contain 88 genes, including 61 protein-coding genes (three of which are duplicated in the IR), four ribosomal RNA genes, and 23 tRNA genes (five of which are duplicated in the IR). Among these genes, six genes (*atpF*, *clpP*, *petB*, *petD*, *rpoC1*, *rpl2*) harboured a single intron and two genes (*ycf3*, *rps12*) had two introns. The gene *rps12* has trans-splicing. The overall A/T content of the plastome was 62.7%, while the corresponding values of the LSC, SSC and IR regions were 65.3%, 73.6%, and 57.1%, respectively.

We used RAxML (Stamatakis 2006) with 1000 bootstraps under the GTRGAMMAI substitution model to reconstruct a maximum likelihood (ML) phylogeny of nine published complete plastomes of Santalales, using *Tetragonia tetragonioides* (Aizoaceae, Caryophyllales) as an outgroup. The phylogenetic analysis indicated that *S. notothixoides* and *Taxillus sutchuenensis* are closely related and all members of Santalales were clustered with a high bootstrap support (BS) value (Figure 1). The *S. notothixoides plastome* reported here will provide a useful resource for the development of medicinal and edible value as well as for phylogenetic studies of Santalales.

**Figure 1.**
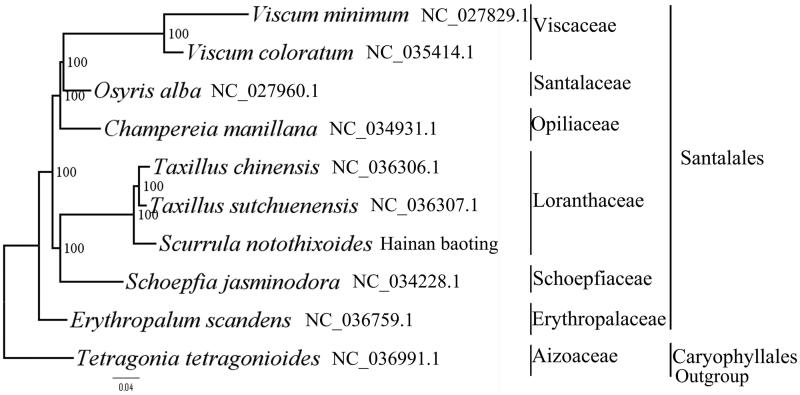
The best ML phylogeny recovered from 10 complete plastome sequences by RAxML. Accession numbers: *Scurrula notothixoides* (this study GenBank Accession number: MH220878), *Taxillus sutchuenensis* NC_036307.1, *Taxillus chinensis* NC_036306.1, *Viscum coloratum* NC_035414.1, *Viscum minimum* NC_027829.1, *Champereia manillana* NC_034931.1, *Schoepfia jasminodora* NC_034228.1, *Erythropalum scandens* NC_036759.1, *Osyris alba* NC_027960.1 and *Tetragonia tetragonioides* NC_036991.1.
